# Fatal sepsis by *Bacillus circulans* in an immunocompromised patient

**Published:** 2011-09

**Authors:** M Alebouyeh, P Gooran Orimi, M Azimi-rad, M Tajbakhsh, E Tajeddin, S Jahani Sherafat, E Nazemalhosseini mojarad, MR Zali

**Affiliations:** Research Center for Gastroenterology and Liver Diseases, Shahid Beheshti University of Medical Sciences, Tehran, Iran.

## Abstract

An immunosuppressed man was admitted to hospital with diarrhea and a history of urinary tract infection. He was subjected to treatment with antibiotics. The patient died of putative severe sepsis. The etiological agent was a carbapenemase producing isolate of *Bacillus circulans* with resistance to all prescribed antimicrobial agents.

## INTRODUCTION

*Bacillus* species are gram positive or gram variable spore forming rods. Most members of *Bacillus* species are distributed in the natural environment, but some of these species are able to cause severe to self-limited disorders as an actual or opportunistic pathogen. Resistance of their spores to UV, disinfectants and some other sterilizing agents have results into that some generated them intended act as contaminants in operating rooms. They may be part of normal flora, particularly in patients hospitalized for prolonged periods. As *Bacillus* spp. are common laboratory contaminants and have been associated with pseudoepidemics in clinics, the initial report of a blood culture growing a *Bacillus* spp. may cause be to ignoringed them in treatment strategies. The present study reports a fatal case of infection with *B. circulans*. The bacterium was isolated from blood culture and was resistant to all of the prescribed antibiotics.

## CASE REPORT

A 62 year-old patient with 4 years of unknown end-stage renal disease, which had a history of kidney graft surgery 11 months ago was admitted to hospital for UTI. The patient was subjected to the treatment regime of sandimmone, cellcept and predinosolone after surgery. He suffered from painless diarrhea 3 weeks before admission and had a history of abdominal pain in lower abdomen. In clinical examination; there was no sign of fever and icter with a brief pale and right lower quadrant tenderness on the side of the grafted kidney without guarding. The pulses were normal. Laboratory results showed an increase in WBC count (11,000 cells per cmm), anemia, HGH (9 ng/ml), BUN (56 mg/dL) and creatinin (2.8 mg/dL). The patient was subjected to therapy for pseudomembraneous colitis with oral vancomycin. Rectosigmoidoscopy was normal. Hydrocortisone was administered with suspicion to graft rejection. At day three, there were signs of hypothermia, tachypnea and increased abdominal pain. Based on symptoms, sepsis was suspected. The patient was subjected to antibiotic therapy (metronidazole and piperacillin- tazobactam). The patient had increased ALP (1900 IU/L) and Bilirubin titers (direct: 16 and total: 30 mg/dL). Increased gall bladder wall thickness and ascites also were observed in sonography. The patient, moreover, has shown elevated PT, PTT, INR and platelet count that could potentially somewhat explain occurrence of DIC; the patient was subjected to fresh frozen plasma therapy and finally expired.

**Fig. 1:**
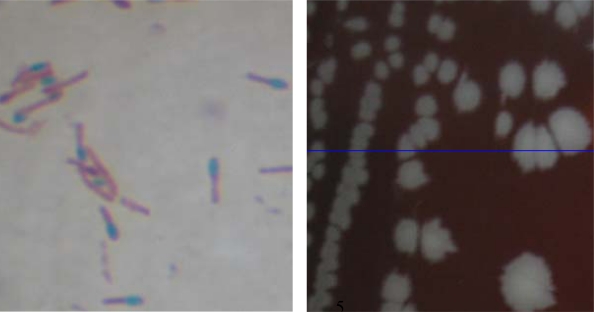
Spore staining and colony morphology of *B. circulans*a.a The bacterium was cultured on sheep blood agar and stained by malachite green and safranin. 100 X.

Blood culture was done to detect the probable responsible microorganism. Results showed a single type of bacterial colony on blood agar. The bacterium was a member of the non-hemolytic spore forming gram positive and strictly anaerobic bacilli (Fig. 1). Morphological and biochemical tests were done according to the standard identification guideline (Table 1). Antimicrobial susceptibility testing was performed using the disk diffusion method (13) (Table 2). To identify the organism, 16S rRNA gene was sequenced (1) and the sequence has been deposited in the GenBank sequence database under accession number HQ315829.

**Table 1:** Identification tests for *Bacillus* species.

Acid from:	
Glucose	+
Xylose	+
Arabinose	+
Mannitol	+
Motility	+
Indol	–
VP	–
Starch	+
Casein	+
NaCl ( 5% and 7%)	+
Catalase	+
Nitrate	–
Growth at 50 ^o^ C	–
Anaerobic growth	+
Hemolysis	–

+ positive, – negative

**Table 2:** Antimicrobial susceptibility of *Bacillus circulans*.

Antimicrobial agent	Disk content (g/ml)	Zone diameter (mm)	Result
Mer	10 g	7	R
Mer-EDTA	10/750 g	18	S
Pen	10 unit	15	R
Amp	10 g	30	S
Ceft	30 g	0	R
Tet	30 g	28	S
Gen	10 g	25	S
Kan	30 g	25	S
Cef	30 g	0	R
Azt	30 g	35	S
Van	30 g	0	R
Pip	75 g	15	R
Met	5 g	0	R
Pip-taz	100/10 g	12	R

Several reports have noticed the importance *of Bacillus* spp. as putative pathogens (*B. subtilis*, *B. coagulans*, *B. pumilis*, *B. sphericus* (2–5). *Bacillus circulans* has been isolated from cases of meningitis (6), cerebrospinal fluid shunt infection (7), prosthetic heart valve (8), endocarditis (9), endophtalmitis (10), wound infection in a patient with malignant ovarian carcinoma (11) and proximal interphalangeal joint infection (12). There are some other reports indicating involvement of this bacterium as a causative agent of bacteremia and sepsis in hospitalized patients. Most patients in these reports were immunocompromised**. Immune-compromised patients may be infected with these bacteria and may suffer from sepsis and other related complications. These bacteria are facultative anaerobe and can multiply in blood and host tissues in the absence of effective immune responses. Disseminated intravascular coagulation (DIC) in some cases is related to bacterial infections, but understanding of the direct impact of bacteria in this phenomenon needs more detailed study.

Sepsis is a systemic inflammatory response to an infection that causes damages to vital body organs such as heart, lungs, kidneys and livers. Sepsis is one of the leading causes of death in hospitalized patients with underlying diseases. Responsible bacteria for sepsis are members of common bacteria residing in hospital environment that are usually resistant to broad spectrum of antibiotics. Results obtained from this study, consistent with other reports, emphasize that *B. circulans* could be considered as a potent pathogen for immunocompromised patients and laboratories should not assume this bacterium as a contaminant. The isolated bacterium was the only bacterial species has been identified by the microbiological tests. It was resistant to the majority antibiotics that were prescribed during the treatment regimen. The infection was occurred in an immunocompromised patient and the experimentally prescribed treatments were not considered as effective. The results of the present study emphasizes the importance of tracking antimicrobial resistance patterns of suspected bacterial agents in severe infections, particularly in immunocompromised patients. Emergence of -lactamase producing bacteria in the hospital setting is largely studied for gram negative bacteria, but there is very little information for gram-positive bacteria, especially those that are considered as nonpathogen (i.e, *Bacillus* spp.). To our knowledge, this is the first report of bacterial infection caused by carbapenemase producing *Bacillus circulans*. More studies in this regard will help us to better understand how this bacterium has acquired this resistant phenotype.

## References

[1] Weisburg WG, Barns SM, Pelletier DA, Lane DJ (1991). 16-ribosomal DNA amplification for phylogenetic study. J Bacteriol.

[2] Oggioni M R, Pozzi G, Valensin P E, Galieni P, Bigazzi C (1998). Recurrent septicemia in an immunocompromised patient due to probiotic strains of Bacillus subtilis. J Clin Microb.

[3] Richard V, Van der Auwera P, Snoeck R, Daneau D, Meunier F (1988). Nosocomial bacteremia caused by Bacillus species. Eur J Clin Microbiol Infect Dis..

[4] Bentur HN, Dalzell AM, Riordan FAI (2007). Central venous catheter infection with Bacillus pumilus in an immunocompetent child: a case report. Ann Clin Microbiol Antimicrob.

[5] Isaacson P, Jacobs PH, Mackenzie AM, Mathews AW (1976). Pseudotumour of the lung caused by infection with Bacillus sphaericus. J Clin Pathol.

[6] Rosovitz MJ, Voskuil MI, Chambliss  GH, Bacillus, Collier J, Balows A, Sussman M (1998). Topley and Wilson's Microbiology and Microbial Infections.9th ed.

[7] Roncoroni A, Rivas M, Smayevsky J, Bianchini H, Zucarro G (1985). Infection of a cerebrospinal fluid shunt system by Bacillus circulans and Bacillus larvae. Rev Argent Microbiol.

[8] Krause A, Gould F. K, Forty J (1999). Prosthetic heart valve endocarditis caused by Bacillus circulans. Journal of Infection.

[9] Gatermann S, Hollandt H, Marre R, Mitusch R, Djonalgic H (1991). Endocarditis caused by Bacillus circulans. Infection..

[10] Tandon A, Tay-Kearney ML, Metcalf C, McAllister L (2001). Bacillus circulansendophthalmitis. Clin Experiment Ophthalmol..

[11] Logan NA, Old D C, Dick HM (1985). Isolation of Bacillus circulans from a wound infection. J Clin Pathol.

[12] Goudswaard WB, Dammer MH, Hol C (1995). Bacillus circulans infection of a proximal interphalangeal joint after a clenched-fist injury caused by human teeth. Eur J Clin Microbiol Infect Dis.

[13] Clinical and Laboratory Standards Institute (2007). M100-S17. Performance standards for antimicrobial susceptibility testin g; 16th informational supplement.

